# Antihypercholesterolemic and Antioxidative Potential of an Extract of the Plant, *Piper betle*, and Its Active Constituent, Eugenol, in Triton WR-1339-Induced Hypercholesterolemia in Experimental Rats

**DOI:** 10.1155/2014/478973

**Published:** 2014-01-09

**Authors:** Karuppasamy Venkadeswaran, Arumugam Ramachandran Muralidharan, Thangaraj Annadurai, Vasanthakumar Vasantha Ruban, Mahalingam Sundararajan, Ramalingam Anandhi, Philip A. Thomas, Pitchairaj Geraldine

**Affiliations:** ^1^Department of Animal Science, School of Life Sciences, Bharathidasan University, Tiruchirappalli, Tamilnadu 620024, India; ^2^Institute of Ophthalmology, Joseph Eye Hospital, Tiruchirappalli, Tamilnadu 620001, India

## Abstract

Hypercholesterolemia is a dominant risk factor for atherosclerosis and cardiovascular diseases. In the present study, the putative antihypercholesterolemic and antioxidative properties of an ethanolic extract of *Piper betle* and of its active constituent, eugenol, were evaluated in experimental hypercholesterolemia induced by a single intraperitoneal injection of Triton WR-1339 (300 mg/kg b.wt) in Wistar rats. Saline-treated hypercholesterolemic rats revealed significantly higher mean blood/serum levels of glucose, total cholesterol, triglycerides, low density and very low density lipoprotein cholesterol, and of serum hepatic marker enzymes; in addition, significantly lower mean serum levels of high density lipoprotein cholesterol and significantly lower mean activities of enzymatic antioxidants and nonenzymatic antioxidants were noted in hepatic tissue samples from saline-treated hypercholesterolemic rats, compared to controls. However, in hypercholesterolemic rats receiving the *Piper betle* extract (500 mg/kg b.wt) or eugenol (5 mg/kg b.wt) for seven days orally, all these parameters were significantly better than those in saline-treated hypercholesterolemic rats. The hypercholesterolemia-ameliorating effect was better defined in eugenol-treated than in *Piper betle* extract-treated rats, being as effective as that of the standard lipid-lowering drug, lovastatin (10 mg/kg b.wt). These results suggest that eugenol, an active constituent of the *Piper betle* extract, possesses antihypercholesterolemic and other activities in experimental hypercholesterolemic Wistar rats.

## 1. Introduction

Hypercholesterolemia is a major socioeconomic problem in common individuals as well as health professionals due to the strong correlation between cardiovascular diseases and lipid abnormalities [1]. The modern lifestyle, with a high fat diet and little physical activity, significantly contributes to hypercholesterolemia and cardiovascular diseases [2]. High levels of low-density lipoprotein (LDL) cholesterol accumulate in the extracellular subendothelial space of arteries; these are highly atherogenic and toxic to vascular cells, leading to atherosclerosis, hypertension, obesity, diabetes, and functional depression in organs such as the liver, heart, and kidneys [3]. Clinical trials have shown that lowering lipids reduces the morbidity and mortality associated with cardiovascular complications [4]. Intensive reduction of LDL-cholesterol levels have also been found to reverse atherosclerosis and decrease the progression of cardiovascular disease [5, 6].

Oxidative stress induced by reactive oxygen species (ROS) plays an important role in the etiology of several diseases, including atherosclerosis and coronary heart disease [7, 8]. Oxidative stress contributes to the development of atherosclerosis in the vascular wall through the formation of ROS [6]. Increased formation of free radicals is accompanied by perturbations in antioxidant status, resulting in oxidative damage to cellular components [8]. Hypercholesterolemia is reported to be associated with the oxidative stress that results from the increased production of ROS or impairment of the antioxidant system [9]. This has nurtured research interest in evaluating antioxidant-rich traditional remedies and alternative medicines as potentially efficacious cholesterol-lowering therapies which have few, or no, side-effects.

The* Piper betle* plant is widely grown in the tropical humid climate of South East Asia and its leaves, with a strong pungent and aromatic flavour, are widely consumed as a mouth freshener. The leaves of *Piper betle,* which are reported to possess medicinal properties, have been widely used as a traditional medicine for treating several diseases including, catarrhal and pulmonary infections [10]. In a preliminary study by Koff et al. [11], an extract of *Piper betle* leaves was found to contain several bioactive molecules such as polyphenols, alkaloids, steroids, saponins, and tannins. More recently, an extract of *Piper betle* was reported to exhibit biological capabilities of detoxification and antioxidative and antimutagenic activities [12]. Eugenol (4-allyl-1-hydroxy-2-methoxybenzene), a natural food-flavouring agent found in plant extracts of *Piper betle*, cinnamon, clove, basil, and nutmeg, has been found to ameliorate oxidative stress by preventing oxidative tissue damage in different experimental models [1, 13, 14]. The efficacy of eugenol was found in human beings to be within the permitted levels by Food and Agricultural Organization/World Health Organization Expert Committee on Food Additives (maximum permitted intake is 2.5 mg/100 g) [15]. Eugenol is reported to be present in a concentration of 0.32% in *Piper betle* [16]; conceivably, this concentration may suffice to exert antioxidant activity. Although *Piper betle* leaves have been reported to possess antioxidative, antifungal, hypotensive, respiratory, antidepressant, antihelminthic, and antibacterial activities [17], there is currently no information on its putative antihyperlipidemic or antiatherogenic potential.

In the present investigation, an attempt has been made to determine whether an ethanolic extract of *Piper betle* and its active constituent, eugenol, possess putative serum lipid-lowering and antioxidant activities in comparison with that of lovastatin (a commercially available serum lipid-lowering drug) in an experimental animal model of hypercholesterolemia.

## 2. Materials and Methods

### 2.1. Chemicals

Lovastatin and eugenol (98%) were purchased from Sigma Chemical Co. (St. Louis, MO, USA). Triton WR-1339 and all the other chemicals and reagents used were of analytical grade and were obtained from Himedia Laboratories (Mumbai, India).

### 2.2. Experimental Animals

Male albino rats of the Wistar strain (150–200 g) were housed under conditions of controlled temperature (25 ± 2°C) with a 12 h/12 h day-night cycle, during which time they had free access to food and water *ad libitum*. Animals were maintained per national guidelines and protocols approved by the Institutional Animal Ethical Committee (BDU/IAEC/58/2013/09.04.2013).

### 2.3. Preparation of Plant Extract

The air-dried leaves of *Piper betle *variety (Bangala Pan) (100 g) were chopped into fine pieces. These were extracted in 95% ethanol (1 L) by using a Soxhlet's apparatus for 72 hours. Extracts were then filtered through Whatman filter paper (no. 1). The solvent was evaporated under reduced pressure at 45° by using a rotary evaporator for elimination of ethanol, and the dried extract was stored at 4°C until further use.

### 2.4. Experimental Induction of Hypercholesterolemia

Hypercholesterolemia was induced experimentally in 12 h-fasted rats by a single intraperitoneal injection of Triton WR-1339 (300 mg/kg body weight (b.wt.)) dissolved in 0.89% saline [18]. Forty-eight hours after administration of Triton WR-1339, rats exhibited elevated serum levels of total cholesterol and triglycerides; these rats were deemed to be hypercholesterolemic and used for further investigation.

### 2.5. Experimental Design

#### 2.5.1. Treatment Groups

The experimental rats were divided into five treatment groups, each comprising five rats. 

*Group I.*  Control rats (not hypercholesterolemic and did not receive any treatment). 

*Group II.* Hypercholesterolemic rats that received only saline orally for 7 days.

*Group III.* Hypercholesterolemic rats that received lovastatin (10 mg/kg b.wt./day) in an aqueous suspension orally for 7 days. 

*Group IV.*  Hypercholesterolemic rats that received the *Piper betle *extract (500 mg/kg b.wt./day) in an aqueous suspension orally for 7 days.

*Group V.*  Hypercholesterolemic rats that received eugenol (5 mg/kg b.wt./day) in 0.5% peanut oil orally for 7 days. 

Saline, lovastatin, *Piper betle* extract, and eugenol were administered orally by gastric intubation once daily for 7 days. Blood samples were collected from all experimental rats on day 10 (7 days after start of treatment), and, subsequently, serum was separated for subsequent analysis of serum lipid profile parameters and serum hepatic marker enzymes. After collection of the blood samples, all the animals were sacrificed by cervical decapitation; from each animal, the liver was excised and stored at −80°C until subsequent analysis of antioxidant activity and the rate of lipid peroxidation in hepatic tissue samples.

#### 2.5.2. Preparation of Hepatic Tissue Samples for Analysis

Hepatic tissue (100 mg tissue/mL buffer) was first homogenized in 50 mM phosphate buffer (pH 7.2); the homogenate was then centrifuged at 12,000 ×g for 15 mins and the supernatant was used for analysis. The protein concentration in each fraction was determined by the method of Bradford [19], using crystalline bovine serum albumin as a standard.

### 2.6. Parameters Analysed

#### 2.6.1. Blood Glucose Level and Serum Lipid Profile Parameters

Mean levels of blood glucose were measured by the method of Sasaki et al. [20]. In the same samples, mean levels of total cholesterol, triglycerides, and high-density lipoprotein (HDL) cholesterol were determined by standard kits (BioSystems, Spain) following the manufacturer's instructions. The atherogenic index (AI) was calculated as AI = (total cholesterol − HDL)/HDL. The levels of LDL cholesterol and very low-density lipoprotein (VLDL) cholesterol were calculated by Friedewald's formula [21], the units being expressed as milligrams per decilitre (mg/dL).

#### 2.6.2. Activities of Hepatic Marker Enzymes in Serum Samples

Activities of aspartate aminotransferase (AST) and alanine aminotransferase (ALT) were determined by the method of King [22] and expressed in terms of micromoles of pyruvate liberated per minute per milligram of protein at 37°C. Alkaline phosphatase (ALP) activity was assayed using disodium phenyl phosphate as substrate [23] and expressed as micromoles of phenol liberated per minute per milligram of protein. Lactate dehydrogenase (LDH) was assayed by the method of King, [24], the principle which is that LDH converts lactate to pyruvate (aided by coenzyme nicotinamide adenine dinucleotide (NAD)), and pyruvate formed reacts with dinitrophenylhydrazine in HCl to yield an orange-colored hydrazone complex in alkaline medium, which is measured at 420 nm.

#### 2.6.3. Activities of Antioxidant Enzymes in Hepatic Tissue Samples

The activities of the antioxidant enzymes catalase (CAT), superoxide dismutase (SOD), glutathione peroxidase (Gpx), and glutathione-S-transferase (GST) were determined by standard methods.

*CAT.* CAT activity was determined by the method of Sinha [25], the principle which is that dichromatic acetic acid is reduced to chromic acetate when heated in the presence of hydrogen peroxide (H_2_O_2_), with the formation of perchloric acid as an unstable intermediate. The resulting green colour was read at 590 nm against a suitable blank on a spectrophotometer. CAT activity was expressed as units per milligram protein (one unit was the amount of enzyme that utilized 1 *µ*mol of H_2_O_2_/min). 

*SOD.* SOD activity (expressed as units/mg protein) was determined by the method of S. Marklund and G. Marklund [26], wherein the degree of inhibition of pyrogallol auto-oxidation by the sample was measured with the change in absorbance being read at 470 nm against blank every minute for 3min on a spectrophotometer. The enzyme activity was expressed as units/mg protein. 

*Gpx.* The activity of Gpx was determined essentially as described by Rotruck et al. [27], wherein the rate at which glutathione is oxidised by H_2_O_2_ (as catalysed by Gpx present in the sample) is determined by reading the colour developed at 412 nm on a spectrophotometer. Gpx activity was expressed as units per milligram protein (one unit being the amount of enzyme that converted 1 *μ*mol of reduced glutathione (GSH) to the oxidized form of glutathione (GSSG) in the presence of H_2_O_2_/min). 

*GST.* The activity of GST was determined by the method of Habig and Jakoby [28], the principle of which is that GSH conjugates with 1-chloro-2,4-dinitrobenzene (c-DNB; a hydrophilic substrate) which is measured spectrophotometrically at 340 nm. GST activity was expressed as *µ*moles of c-DNB formed/min/mg of protein.

#### 2.6.4. Levels of Nonenzymatic Antioxidants (GSH, Ascorbic Acid, and *α*-Tocopherol) in Hepatic Tissue Samples

*GSH*. GSH content (*µ*g/mg protein) was estimated by the method of Moron et al. [29], wherein protein in the sample is first precipitated out, followed by addition 4 mL of 0.3 M Na_2_HPO_4_ (pH 8.0) and 0.5 mL of 0.04% (w/v) 5,5-dithiobis-2-nitrobenzoic acid to the protein-free supernatant to yield a yellow colour that is read spectrophotometrically at 412 nm.

*Ascorbic Acid (Vitamin C).* Vitamin C (*µ*g/mg protein) was measured by the method of Omaye et al. [30], wherein ascorbate in the sample is oxidized by copper to form dehydroascorbic acid which reacts with 2,4-dinitrophenyl hydrazine to form bis-2,4-dinitrophenyl hydrazine which, in turn, undergoes further rearrangement to form a product with an absorption maximum at 520 nm.

**α*-Tocopherol (Vitamin E).* Vitamin E (*µ*g/mg protein) was estimated by the method of Desai [31], the principle which is that ferric ions are reduced to ferrous ions in the presence of tocopherol, resulting in the formation of a pink colour that is read spectrophotometrically at 536 nm.

#### 2.6.5. Determination of Lipid Peroxidation in Hepatic Tissues

The mean concentration of malondialdehyde (MDA), a measure of lipid peroxidation, was assayed in the form of thiobarbituric acid-reacting substances (TBARS) by the method of Ohkawa et al. [32]. Briefly, to 0.2 mL of 8.1% sodium dodecyl sulphate, 1.5 mL of 20% acetic acid (pH 3.5) and 1.5 mL of 0.81% thiobarbituric acid aqueous solution were added in succession. To this reaction mixture, 0.2 mL of the homogenate of hepatic tissue was added. The mixture was then heated in a boiling water bath for 60 min. After cooling to room temperature, 5 mL of butanol : pyridine (15 : 1, v/v) solutions were added. The mixture was then centrifuged at 2000 ×g for 15 min. The upper organic layer was separated, and the intensity of the resulting pink colour was read at 532 nm. Tetramethoxypropane was used as an external standard. The level of lipid peroxides was expressed as nmoles of MDA formed/mg protein.

#### 2.6.6. Histopathological Studies

Conventional techniques of paraffin-wax sectioning and haematoxylin-eosin (HE) staining were used for histological studies [33]. Slices of fresh hepatic tissue were cut and fixed in buffered neutral formalin fixative for 24 h. Following fixation, the tissue slices were washed and processed through an ascending series of alcohol (30%, 50%, 70%, 90%, and 100%), cleared in methyl salicylate, and infiltrated with wax at 57°C. The hepatic tissue, thus cleared, was embedded in paraffin. Sections of 6–8 *µ*m thickness were cut, stained by aqueous haematoxylin and alcoholic-eosin, and then examined by bright-field microscopy (200x) (Carl Zeiss Axioskop 2 plus; Jena, Germany).

#### 2.6.7. Statistical Analysis

The values are expressed as mean ± standard deviation (SD) for five animals in each group. Differences between groups were assessed by one-way analysis of variance (ANOVA) using Statistical Package for Social Sciences software package for Windows (version 16.0; IBM Corp., Armonk, NY, USA). If one-way ANOVA yielded significant results, post hoc testing was performed for intergroup comparisons using the least significant difference test. Values were considered statistically significant when *P* < 0.05.

## 3. Results

### 3.1. Blood Glucose Levels in Wistar Rats (Table 1)

The mean blood glucose level in hypercholesterolemic, saline-treated (group II) rats was significantly (*P* < 0.05) higher than that in control (group I) rats. In hypercholesterolemic rats treated with lovastatin (group III), *Piper betle* extract (group IV), or eugenol (group V), significantly (*P* < 0.05) lower mean blood glucose levels were observed when compared to that in saline-treated hypercholesterolemic rats although the levels were still significantly (*P* < 0.05) higher than that in the control rats. The mean blood glucose level was significantly (*P* < 0.05) higher in *Piper betle* extract-treated hypercholesterolemic rats than that in lovastatin-treated or eugenol-treated hypercholesterolemic rats. However, no significant difference was observed between the mean blood glucose level in lovastatin-treated hypercholesterolemic rats and that in eugenol-treated hypercholesterolemic rats (Table 1).

### 3.2. Serum Lipid Profile Parameters in Wistar Rats (Table 1)

Saline-treated hypercholesterolemic rats showed significantly (*P* < 0.05) higher mean serum levels of total cholesterol, triglycerides, LDL-cholesterol, and VLDL-cholesterol, a significantly higher atherogenic index and a significantly (*P* < 0.05) lower mean level of HDL-cholesterol, when compared to the values in control rats and in lovastatin-treated, *Piper betle* extract-treated, or eugenol-treated hypercholesterolemic rats (Table 1). However, hypercholesterolemic rats treated with lovastatin or *Piper betle *extract exhibited significantly (*P* < 0.05) higher mean serum levels of total cholesterol, triglycerides, LDL-cholesterol, and VLDL-cholesterol, a higher atherogenic index as well as significantly (*P* < 0.05) lower mean serum levels of HDL-cholesterol, when compared to control rats. No significant differences were observed in these parameters between hypercholesterolemic rats that had been treated with *Piper betle* extract or with lovastatin (Table 1). Interestingly, eugenol-treated rats exhibited a significantly (*P* < 0.05) lower mean level of total cholesterol than that in lovastatin-treated rats. In addition, the mean serum total cholesterol, triglyceride, and VLDL-cholesterol levels in eugenol-treated hypercholesterolemic rats were significantly (*P* < 0.05) lower than those observed in *Piper betle *extract-treated hypercholesterolemic rats. A very noteworthy finding was that the mean serum levels of triglycerides and VLDL-cholesterol in eugenol-treated hypercholesterolemic rats approximated those in normal rats (Table 1).

### 3.3. Activities of Hepatic Marker Enzymes in Serum of Wistar Rats (Table 2)

The mean activities of serum AST, ALT, ALP, and LDH were found to be significantly (*P* < 0.05) higher in hypercholesterolemic, saline-treated rats than those in control rats. Although hypercholesterolemic rats treated with the *Piper betle *extract or eugenol exhibited significantly (*P* < 0.05) lower mean activities of these enzymes than those in hypercholesterolemic saline-treated rats, the mean activities of ALT, ALP, and LDH were still significantly higher than those in control rats (Table 2). Interestingly, the mean activity of LDH was significantly (*P* < 0.05) lower in the *Piper betle* extract-treated and in the eugenol-treated hypercholesterolemic rats than those in the lovastatin-treated hypercholesterolemic rats. No significant differences were observed in the mean serum activities of ALT, ALP, and LDH between hypercholesterolemic rats that had been treated with the *Piper betle* extract and those that had been treated with eugenol (Table 2).

### 3.4. Activities of Enzymatic Antioxidants in Hepatic Tissue of Wistar Rats (Table 3)

The mean activities of CAT, SOD, Gpx, and GST in the samples of hepatic tissue from hypercholesterolemic saline-treated rats were significantly (*P* < 0.05) lower than those in control rats (Table 3). In hypercholesterolemic rats that had been treated with lovastatin, *Piper betle* extract, or eugenol, significantly (*P* < 0.05) higher mean activities of these enzymes were noted than those in hypercholesterolemic saline-treated rats; however, these mean enzyme activities remained significantly lower than those in control rats (Table 3). Interestingly, the mean hepatic Gpx activity observed in hypercholesterolemic, eugenol-treated rats was significantly (*P* < 0.05) higher than that in lovastatin-treated hypercholesterolemic rats, while there were no significant differences between these two groups in the mean hepatic activities of CAT and SOD. Also, there were no significant differences in the mean hepatic enzyme activities between hypercholesterolemic* Piper betle* extract-treated and hypercholesterolemic lovastatin-treated rats (Table 3).

### 3.5. Concentrations of Nonenzymatic Antioxidants in Hepatic Tissue of Wistar Rats (Table 3)

The mean concentrations of GSH, vitamin C, and vitamin E in the hepatic tissue samples from hypercholesterolemic, saline-treated rats were significantly (*P* < 0.05) lower than those in control rats (Table 3). However, significantly (*P* < 0.05) higher mean concentrations of these antioxidants were observed in hypercholesterolemic rats that had been treated with lovastatin, *Piper betle* extract, or eugenol than those in hypercholesterolemic, saline-treated rats. There were no significant differences in the mean values of these parameters between hypercholesterolemic rats that had been treated with the *Piper betle *extract and those that had been treated with eugenol. Interestingly, the mean hepatic GSH concentrations in these two groups of rats were found to be significantly (*P* < 0.05) higher than that in the hypercholesterolemic, lovastatin-treated rats. In hypercholesterolemic rats that received eugenol, mean hepatic tissue antioxidant concentrations approached those in control rats; in hypercholesterolemic rats that received the *Piper betle* extract, the mean hepatic vitamin C level approached that in control rats (Table 3).

### 3.6. MDA Concentrations in Hepatic Tissues of Wistar Rats (Table 3)

The mean concentration of MDA in hepatic tissue samples from hypercholesterolemic, saline-treated rats was significantly (*P* < 0.05) higher than that in control rats (Table 3). Although the mean hepatic MDA concentrations in hypercholesterolemic rats that had been treated with lovastatin, *Piper betle* extract, or eugenol were significantly (*P* < 0.05) lower than that in hypercholesterolemic, saline-treated rats, they remained significantly (*P* < 0.05) higher than that in control rats. Hypercholesterolemic rats treated with eugenol exhibited a significantly lower mean concentration of MDA than those treated with the *Piper betle *extract. Interestingly, no significant differences were observed in the mean hepatic tissue concentration of MDA in eugenol-treated or *Piper betle *extract-treated hypercholesterolemic rats, when compared with the mean hepatic tissue concentrations noted in the lovastatin-treated hypercholesterolemic rats.

#### 3.6.1. Histopathological Examination

**Figure 1:**
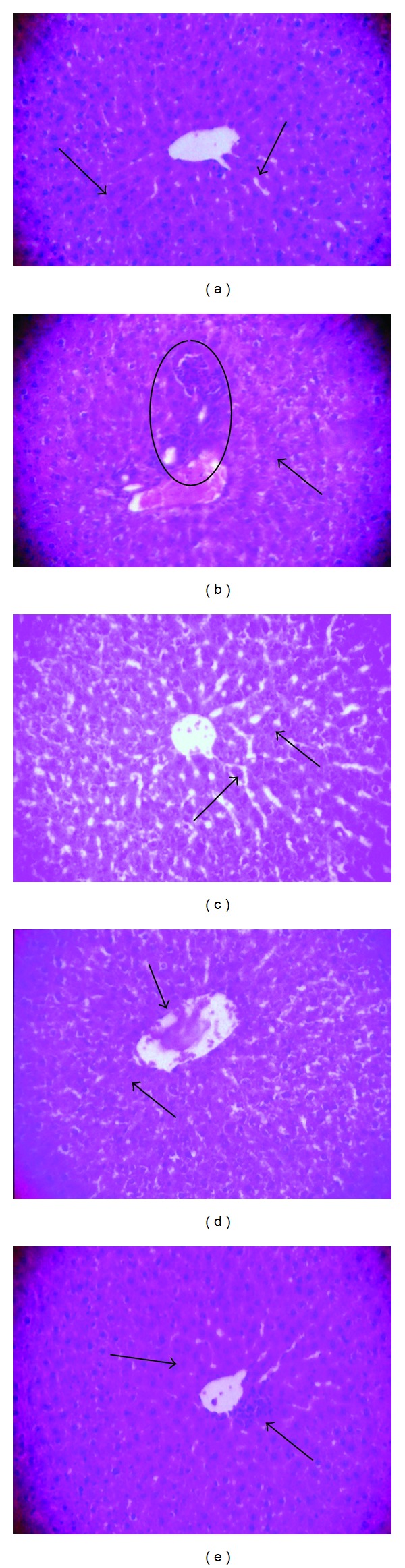
Histoarchitecture of hepatic tissue Wistar rats. Sections of hepatic tissue from the experimental groups of rats were stained by H&E and then subjected to histopathological examination by light microscopy (Figure 1). Sections of hepatic tissue from control rats showing central vein with normal hepatocyte, healthy nucleus, and sinusoidal spaces with kupffer cells (arrows) (a). In sections from hypercholesterolemic saline-treated rats, revealing loss of normal liver radiating pattern, periportal inflammation with cellular infiltration in central vein (Marked place), and vacuolated hepatocytes (arrows) with the nucleus pushed to periphery (b). In hypercholesterolemic lovastatin-treated rats, section showed normal hepatocyte with darkly stained nucleus, (arrows) central vein and wide sinusoids (c). In hypercholesterolemic *Piper betle *extract-treated rats, section showed illustrating few small vacuolated hepatocytes with occasional inflammatory cell infiltration (d). In hypercholesterolemic eugenol-treated rats, sections showed normal hepatic architecture, with parenchymal structures preserved (e).

Sections of hepatic tissue from the experimental groups of rats were stained by H&E and then subjected to histopathological examination by light microscopy (Figure 1). Sections of hepatic tissue from control rats exhibited normal hepatocytes, with normal nuclei and sinusoidal spaces with Kupffer cells (arrows) (Figure 1(a)). In sections from hypercholesterolemic saline-treated rats, revealing loss of normal liver radiating pattern, periportal inflammation with cellular infilteration in central vein (detached line), vacuolated hepatocytes (arrows) with the nucleus pushed to periphery (Figure 1(b)). In hypercholesterolemic lovastatin-treated rats, section showed normal hepatocyte with darkly stained nucleus, (arrows) central vein, and wide sinusoids (Figure 1(c)). In hypercholesterolemic *Piper betle* extract-treated rats, section showed illustrating few small vacuolated hepatocytes with occasional inflammatory cell infilteration (Figure 1(d)). In hypercholesterolemic eugenol-treated rats, sections showed normal hepatic architecture, with parenchymal structures preserved (Figure 1(e)).

## 4. Discussion

Triton WR-1339, one of the well-known non-ionic detergent (oxyethylated tertiary octylphenol formaldehyde polymer), that has been widely used to produce acute hyperlipidemia in animal models. Triton WR-1339-induced hypercholesterolemia has been demonstrated to alter the physicochemical properties of lipoproteins, thereby preventing the uptake of lipoproteins from the circulation through extra hepatic tissues resulting in increased level of circulatory lipoproteins in animal models [34]. Triton WR-1339 model being a rapid and convenient system [18] has been extremely employed for screening natural [35–37] or chemical hypolipidemic drugs [38–40] and also to delineate features of cholesterol and triacylglycerol metabolism [41]. In addition, Triton WR-1339 has also been used successfully to study intestinal lipoprotein synthesis in animal models [42]. Hence, the Triton WR-1339 model has been examined not only as a screening method for antihyperlipidemic agents, but also as a means for elucidating lipid metabolism [43]. In the present study, the putative antihypercholesterolemic effects of an ethanol extract of *Piper betle* and of one of its active constituents, eugenol, were compared with those of a well-known lipid-lowering drug, lovastatin, in a rodent model of hypercholesterolemia that was induced by Triton WR-1339.

Hyperglycemia and hyperlipidemia are important risk factors for diabetes-accelerated atherosclerosis. The main aim of treatment in patients with hyperlipidemia is to reduce the risk of developing ischemic heart disease or the occurrence of further cardiovascular or cerebrovascular complications [44]. Since hyperlipidemia is frequently associated with hyperglycemia, an attempt was made in the current study to measure the blood glucose level in hypercholesterolemic rats (Table 1). In hypercholesterolemic saline-treated rats, a significantly higher mean blood glucose level than that in control (normal) rats was noted (Table 1). However, the administration of the *Piper betle* extract or eugenol significantly decreased the blood glucose levels in hypercholesterolemic rats. These results are consistent with those of an earlier study in which an extract of the flower of *Cassia auriculata* significantly decreased serum glucose levels in hyperlipidemic rats [37].

In the present investigation, in hypercholesterolemic rats that had received the *Piper betle *extract or eugenol, significantly lower mean serum levels of total cholesterol, triglycerides, LDL-cholesterol and VLDL-cholesterol and significantly higher mean serum levels of HDL-cholesterol than those in hypercholesterolemic saline-treated rats were noted. This effect was more pronounced in rats that had received eugenol than in rats that had received the extract of* Piper betle*. The lipid-lowering effect of leaves of *Piper betle* and of its active component, eugenol, in an experimental animal model of hypercholesterolemia is probably mediated through inhibition of hepatic cholesterol biosynthesis and reduction of lipid absorption in the intestine. In the present study, the lower mean serum total cholesterol levels were associated with lower levels of the LDL-cholesterol fraction. Serum LDL-cholesterol level is a major and potentially modifiable risk factor of cardiovascular diseases and serves as a target for numerous antihypercholesterolemic therapies. Our finding suggests that the cholesterol-lowering activity of the *Piper betle* extract is due to enhanced catabolism of LDL-cholesterol through hepatic receptors, as suggested by Khanna et al. [43]. In addition, higher mean serum levels of HDL-cholesterol were noted, which is reported to have a preventive function against atherogenesis. An independent inverse relationship between blood HDL-cholesterol levels and cardiovascular risk incidence has been documented and reported [45]. HDL-cholesterol is commonly termed as “good cholesterol,” since it facilitates the mobilisation of triglycerides and cholesterol from plasma to liver, where these are catabolised and eliminated in the form of bile acids.

Elevated plasma levels of triglycerides are found to be associated with an increased incidence of coronary artery disease [46]. Such higher plasma triglyceride levels have been attributed mainly to an increased population of small, dense LDL-cholesterol deposits which are very atherogenic [47] and enhanced cholesteryl ester mass transfer from apolipoprotein B-containing lipoproteins (VLDL-cholesterol and LDL-cholesterol) [4]. Triglycerides are also proposed to be a major determinant of cholesterol esterification, its transfer, and HDL-cholesterol remodelling in human plasma [48]. The restoration of catabolic metabolism of triglycerides could be due to an increased stimulation of the lipolytic activity of plasma lipoprotein lipase. These alterations in the levels of serum lipid peroxide and antioxidant status in subjects with high serum total cholesterol and LDL-cholesterol levels and low HDL-cholesterol levels may increase the susceptibility of LDL-cholesterol to oxidation in the circulation.

As enhanced lipid peroxidation leads to higher atherogenicity, it is plausible that antioxidant status should have a major impact not only on the rate of LDL oxidation but perhaps on development of atherosclerosis [49]. A potential risk of atherosclerosis in individuals with high serum lipid levels may be associated with LDL oxidation as a result of increased levels of LDL-cholesterol and decreased antioxidant enzyme activity. In the present study, administration of an extract of *Piper betle*, or of eugenol, to hypercholesterolemic rats resulted in significantly lower mean serum triglyceride levels than the mean level seen in hypercholesterolemic, saline-treated rats (Table 1). This effect may have been due to enhanced catabolism of triglycerides caused by increased stimulation of plasma lipoprotein lipase activity. Higher mean levels of HDL-cholesterol were also noted in hypercholesterolemic rats that had been treated with lovastatin, *Piper betle* extract, or eugenol, when compared to the mean level in hypercholesterolemic, saline-treated rats. The lipid-lowering effect brought about by administration of the *Piper betle* extract and of eugenol might have been due to reactivation of lipolytic enzymes for early clearance of lipids from the circulation in triton-induced hyperlipidemia. Our results are consistent with those of Vallianou et al. [50].

The atherogenic index (ratio of LDL-cholesterol to HDL-cholesterol) is also a predictive indicator of cardiovascular disease incidence [35]. Apparently, lowering the atherogenic index is an important measure in reducing the risk of atherosclerosis. In the present study, hypercholesterolemic rats that had been administered *Piper betle* extract or eugenol exhibited significantly lower mean atherogenic index values than did hypercholesterolemic, saline-treated rats.

Küçükgergin et al. [51] demonstrated that hypercholesterolemia is a primary factor contributing to oxidative damage to hepatocytes, leading to malfunctioning of the liver through microvesicular steatosis and intracellular lipid accumulation. The extent of hepatic damage can be assessed by noting the mean activities of serum transaminases and alkaline phosphatase (ALP) [52]. In the present study, the mean activities of serum AST, ALT, ALP, and LDH were significantly higher in hypercholesterolemic, saline-treated rats than those in control rats (Table 2). However, such elevations in the mean levels of serum AST, ALT, ALP, and LDH enzymes appear to have been prevented in hypercholesterolemic rats that had been treated with the *Piper betle* extract or with eugenol, since the mean levels were significantly lower than those in hypercholesterolemic, saline-treated rats (Table 2); these observations suggest that the *Piper betle* extract and eugenol were able to protect the hepatic tissue from hypercholesterolemia-induced oxidative stress-mediated cellular damage. These results are consistent with those of an earlier study, in which the mean serum levels of AST, ALT, ALP, and LDH were found to be significantly lower in rats with Triton WR-1339-induced acute hypercholesterolemia that had been treated with a mushroom extract or with chrysin [53].

Oxygen-free radicals are found to be produced during hypercholesterolemic atherogenesis [54]. Living tissues are endowed with innate antioxidant defense mechanisms through enzymatic and nonenzymatic antioxidants that are involved in the quenching of superoxide anions and H_2_O_2_ [55]. A reduction in the activity of these enzymes is associated with the accumulation of highly reactive free radicals, leading to deleterious effects such as loss of integrity and function of cell membranes [56]. In the present investigation, the mean activities of CAT, SOD, GPx, and GST in hepatic tissue samples from hypercholesterolemic saline-treated rats were significantly (*P* < 0.05) lower than those noted in control rats. However, such a decline in the mean activities of CAT, SOD, GPx, and GST appears to have been prevented in hepatic tissue sample from hypercholesterolemic rats that had been treated with lovastatin, the *Piper betle *extract or eugenol, since the mean activities were significantly higher than those in samples from hypercholesterolemic, saline-treated rats (Table 3). The antioxidant activity of eugenol, which has a phenolic structure, has already been evaluated by the extent of protection offered against free radical-mediated lipid peroxidation in both *in vitro* and *in vivo* studies [13].

Nonenzymatic antioxidants also play a vital role in protecting cells from oxidative damage. GSH is an important antioxidant in living systems because it is involved in numerous biochemical pathways within the cells. It plays a key role in liver detoxification reactions by maintaining the structural integrity of cell membranes [57]. *α*-tocopherol (Vitamin E), a nonenzymatic antioxidant, is believed to protect biological membranes from oxidative damage by its ability to quench lipid peroxides [58]. It is possible that elevated levels of oxygen-free radicals in hypercholesterolemia may damage the myocardial cell besides affecting the coronary arteries. Ascorbic acid (Vitamin C), the most widely recognized water-soluble antioxidant, prevents the oxidative damage to the cell membrane that is induced by aqueous radicals; it also reduces and regenerates oxidized *α*-tocopherol and lipid peroxides [59]. In the present study, the mean hepatic concentrations of GSH and of vitamins C and E were found to be significantly lower in hypercholesterolemic, saline-treated rats than those in control rats (Table 3), possibly due to lipidemic oxidative stress. However, the mean hepatic concentrations of GSH and of vitamins C and E were significantly higher in hypercholesterolemic rats that had been treated with lovastatin, *Piper betle* extract, or eugenol than those in hypercholesterolemic, saline-treated rats (Table 3). No significant differences were noted in these test parameters between lovastatin-treated, *Piper betle *extract-treated, and eugenol-treated hypercholesterolemic rats. Hence, treatment with the *Piper betle* extract or eugenol possibly acted by reducing lipidemic oxidative stress, therein permitting these antioxidants to be maintained at near normal levels.

In biological environments, the most favourable substrate for lipid peroxidation is represented by polyunsaturated fatty acids. Hypercholesterolemia-mediated atherosclerosis is associated with an increase in the level of the lipid peroxidation product, malondialdehyde (MDA), which is an index of the level of oxygen-free radicals [54, 60]; it also reacts with polyunsaturated fatty acids, causing free radical-mediated tissue damage in cellular membranes. The polyunsaturated fatty acids in the cell membrane are protected against lipid peroxidation through endogenous antioxidants such as *α*-tocopherol [61]. A decrease in lipid peroxidation leads to a reduction in arterial wall cholesterol content. Therefore, reduction of atherosclerosis caused by hypercholesterolemia is associated with a decrease in lipid peroxidation, while increased lipid peroxidation is a characteristic feature of hypercholesterolemia; it impairs cell membrane fluidity and alters the activity of membrane-bound enzymes and receptors, resulting in membrane malfunction [55].

Eugenol might be effective in preventing the toxic manifestations produced by increased levels of lipids induced by triton WR-1339. In the present study, the oral administration of eugenol or of the *Piper betle *extract to hypercholesterolemic rats resulted in significantly lower mean levels of MDA than that in saline-treated hypercholesterolemic rats. The decrease in intensity of lipid peroxidation, as inferred from the lower mean levels of MDA, was possibly due to the free radical-scavenging property of the hydroxyl groups at the seventh position of the eugenol molecule.

Hypercholesterolemia-induced hepatic abnormalities can be further confirmed by histopathological findings. In the present investigation, Triton WR-1339-induced hypercholesterolemic rats that had been treated with saline alone showed marked changes in the liver, ballooning degeneration of the hepatocytes, and occasional collection of chronic inflammatory cells (Figure 1). Deepa and Varalakshmi [62] observed similar fatty changes in the hepatic tissue, which are consistent with the abnormal biochemical parameters observed in the present study. However, treatment with eugenol appeared to ameliorate or prevent the adverse effects, as suggested by the presence of only minimal or partial fatty changes. So also Sudhahar et al. [63] reported that the administration of lupeol and lupeol linoleate to hypercholesterolemic rats resulted in reduction of fatty changes in hepatic tissue.

## 5. Conclusion

In conclusion, the present investigation has demonstrated the putative lipid-lowering effect (by virtue of antioxidant activity) of an ethanolic extract of *Piper betle* and of eugenol, the major constituent of the *Piper betle* extract, in Triton WR-1339-induced, hypercholesterolemic rats. The lipid-lowering potential and antioxidant capacity of eugenol appeared to be more pronounced than that of the *Piper betle* extract and as effective as that of the standard lipid-lowering drug, lovastatin. Hence, eugenol may possibly be developed as an alternative cholesterol-lowering drug; however, further molecular studies are required to investigate the mechanism underlying the antihypercholesterolemic effect of this compound. Future studies must focus on the hypolipidemic effect of eugenol under conditions of chronic hypercholesterolemia.

## Figures and Tables

**Table 1:** Mean levels of blood glucose and of serum lipid profile parameters* in Wistar rats.

Parameters tested	Group I (control)	Group II hypercholesterolemic, saline-treated	Group III hypercholesterolemic, lovastatin-treated	Group IV hypercholesterolemic, *Piper betle *extract treated	Group V hypercholesterolemic, eugenol-treated
Glucose	84.5 ± 2.4	194.1 ± 2.1^a^	144.2 ± 1.1^ab^	149.2 ± 0.9^abc^	141.9 ± 1.3^abd^
Total cholesterol	49.2 ± 2.4	134.2 ± 4.7^a^	61.5 ± 1.6^ab^	62.2 ± 2.8^abc^	59.2 ± 3.1^abc^
Triglycerides	44.6 ± 2.4	149.6 ± 2.7^a^	59.7 ± 0.9^ab^	63.7 ± 1.6^abc^	53.4 ± 2.9^abc^
HDL cholesterol	29.4 ± 4.7	20.2 ± 2.1^a^	28.0 ± 0.2^ab^	26.7 ± 0.7^ac^	28.4 ± 4.2^abcd^
LDL cholesterol	13.6 ± 2.4	109.7 ± 0.5^a^	39.4 ± 1.3^ab^	40.2 ± 2.7^ab^	22.5 ± 7.2^acd^
VLDL cholesterol	4.8 ± 6.8	35.2 ± 1.9^a^	12.5 ± 1.0^ab^	14.5 ± 0.2^abc^	11.2 ± 0.5^abd^
A.I.	0.6 ± 0.1	4.8 ± 0.3^a^	2.0 ± 0.3^ab^	2.3 ± 0.2^b^	1.3 ± 0.3^abcd^

*Sampling was done 10 days after induction of hypercholesterolemia and 7 days after start of treatment.

Values represent the mean ± SD for observations made on five rats in each group.

Units: milligrams per deciliter (except for atherogenic index).

Statistical analysis: One-way analysis of variance (ANOVA), where significant, post hoc testing (least significant difference) was done for intergroup comparisons.

HDL-C: high-density lipoprotein cholesterol, LDL-C: low-density lipoprotein cholesterol, VLDL-C: very low-density lipoprotein-cholesterol, A.I.: atherogenic index.

^a^Statistically significant difference (*P* < 0.05) when compared with group I values.

^b^Statistically significant difference (*P* < 0.05) when compared with group II values.

^c^Statistically significant difference (*P* < 0.05) when compared with group III values.

^d^Statistically significant difference (*P* < 0.05) when compared with group IV values.

**Table 2:** Mean serum levels of hepatic marker enzymes in* Wistar rats.

Parameters tested	Group I (control)	Group II hypercholesterolemic, saline treated	Group III hypercholesterolemic, lovastatin treated	Group IV hypercholesterolemic, *Piper betle *extract treated	Group V hypercholesterolemic, eugenol treated
AST	0.8 ± 0.2	1.8 ± 0.2^a^	1.6 ± 0.2^ab^	1.3 ± 0.3^ab^	1.2 ± 0.2^bcd^
ALT	1.2 ± 0.03	1.8 ± 0.3^a^	1.6 ± 0.2^ab^	1.2 ± 0.1^ab^	1.3 ± 0.3^ab^
ALP	2.0 ± 0.1	3.3 ± 0.7^a^	3.0 ± 0.1^a^	3.2 ± 0.1^ab^	2.8 ± 0.3^ab^
LDH	6.9 ± 0.4	17.2 ± 0.5^a^	13.4 ± 0.7^ab^	12.2 ± 0.4^abc^	12.5 ± 0.5^abc^

*Sampling done 10 days after induction of hypercholesterolemia and 7 days after start of treatment.

Values represent the mean ± SD for observations made on five rats in each group.

Units: aspartate and alanine aminotransferases: *µ*moles × 10^−2^ of pyruvate liberated/min/mg protein.

Alkaline phosphatase: *µ*moles × 10^−2^ of phenol liberated/min/mg protein.

Lactate dehydrogenase: *µ*moles × 10^−1^ of pyruvate formed/minute/mg protein.

Statistical analysis: one-way analysis of variance (ANOVA), where significant, post hoc testing (least significant difference) done for intergroup comparisons.

AST: aspartate aminotransferase, ALT: alanine aminotransferase, ALP: alkaline phosphatase, LDH: lactate dehydrogenase.

^a^Statistically significant difference (*P* < 0.05) when compared with group I values.

^b^Statistically significant difference (*P* < 0.05) when compared with group II values.

^c^Statistically significant difference (*P* < 0.05) when compared with group III values.

^d^Statistically significant difference (*P* < 0.05) when compared with group IV values.

**Table 3:** Mean activities of enzymatic antioxidants and mean levels of nonenzymatic antioxidants and malondialdehyde in hepatic tissue samples* from Wistar rats.

Parameters tested	Group I (control)	Group II hypercholesterolemic, saline treated	Group III hypercholesterolemic, lovastatin treated	Group IV hypercholesterolemic, *Piper betle *extract treated	Group V hypercholesterolemic, eugenol treated
SOD	7.1 ± 1.4	4.5 ± 0.5^a^	5.0 ± 0.4^ab^	5.3 ± 0.2^a^	5.5 ± 0.3^abc^
CAT	53.8 ± 3.5	40.1 ± 4.0^a^	42.9 ± 3.2^b^	43.8 ± 0.2^ac^	44.6 ± 5.7^abd^
GPX	31.3 ± 5.5	13.4 ± 1.1^a^	20.8 ± 1.3^ab^	21.5 ± 2.1^ab^	22.4 ± 0.7^ab^
GST	17.0 ± 4.4	8.4 ± 1.0^a^	14.4 ± 1.8^b^	14.5 ± 0.6^abc^	14.7 ± 0.6^abcd^
GSH	3.3 ± 0.1	2.0 ± 0.1^a^	2.6 ± 0.1^ab^	2.7 ± 0.1^ab^	2.8 ± 0.1^abd^
VIT-C	2.3 ± 1.4	1.6 ± 0.6^a^	1.7 ± 0.8^a^	1.9 ± 0.7^ab^	1.8 ± 0.6^abcd^
VIT-E	1.9 ± 1.2	1.0 ± 0.4^a^	1.3 ± 0.6^ab^	1.5 ± 0.1^abc^	1.5 ± 0.7^bcd^
MDA	1.2 ± 0.1	3.8 ± 0.4^a^	1.8 ± 0.3^ab^	2.0 ± 0.2^abc^	1.6 ± 0.1^acd^

*Sampling done 10 days after induction of hypercholesterolemia and 7 days after start of treatment.

Values represent the mean ± SD for observations made on five rats in each group.

Units: CAT—*µ*moles of H_2_O_2_ utilized/min/mg protein.

SOD—units/mg protein.

Gpx—*µ*moles of GSH oxidized/min/mg protein.

GST—*µ*moles of c-DNB formed/min/mg protein.

GSH—microgram of reduced glutathione/mg protein.

Vitamins C and E—micrograms/mg protein.

MDA—*µ*moles of MDA produced/mg protein.

Statistical analysis: one-way analysis of variance (ANOVA), where significant, post hoc testing (least significant difference) done for intergroup comparisons.

CAT: catalase, SOD: superoxide dismutase, Gpx: glutathione peroxidase, GST: glutathione-S-transferase, GSH: reduced glutathione, MDA: malondialdehyde, H_2_O_2_: hydrogen peroxide, c-DNB: 1-chloro-2,4-dinitrobenzene.

^a^Statistically significant difference (*P* < 0.05) when compared with group I values.

^b^Statistically significant difference (*P* < 0.05) when compared with group II values.

^c^Statistically significant difference (*P* < 0.05) when compared with group III values.

^d^Statistically significant difference (*P* < 0.05) when compared with group IV values.
